# Caecal Epithelium‐Derived Thymic Stromal Lymphopoietin is not Required for Protective Immunity Against Whipworm

**DOI:** 10.1002/eji.70248

**Published:** 2026-07-24

**Authors:** Connor P. Lynch, Seona Thompson, Erin‐Claire Pallott, Laura Campbell, Sang Hun Lee, Richard K. Grencis

**Affiliations:** ^1^ Manchester Cell‐Matrix Centre Faculty of Biology, Medicine, and Health Lydia Becker Institute of Immunology and Inflammation University of Manchester Manchester UK; ^2^ Laboratory of Parasitic Diseases National Institute of Allergy and Infectious Diseases National Institutes of Health Bethesda Maryland USA

**Keywords:** biology, conditional gene knockout, cytokine, helminths, immunity, immunology, thymic stromal lymphopoietin, trichuris muris

## Abstract

Recent research examining classical type two alarmin TSLP has uncovered non‐epithelial cellular sources of the cytokine in various murine challenges. *Trichuris muris* is a key model of type two immunity in the intestine, with expulsion being TSLP‐dependent. Previous research has indicated epithelial production of TSLP in the caecum, but its production during infection has not been characterised in detail. Here, conditional knockout of *Tslp* expression in caecal epithelial cells was shown not to impact parasite expulsion, but, surprisingly, neutralisation of TSLP from days 15 to 25 post‐infection produced full susceptibility. qPCR and transcript‐based imaging were used to demonstrate that caecal epithelium is a poor source of *Tslp*, and non‐epithelial *Tslp* expression is observed during *Trichuris* infection in EpCAM‐negative cells. These data build on previous work to raise important questions regarding the interaction of TSLP with immunity broadly, as well as the conditions for the production of immunity to intestinal helminths.

## Graphical abstract

Immunity to *Trichuris muris* infection is unimpacted by epithelial knockout of TSLP expression. Imaging of transcription in the caecum reveals *Tslp* transcription throughout infection in EpCAM‐, Foxl1‐, Ccl24‐ lamina propria‐resident cells, contributing during the expulsion phase of infection.

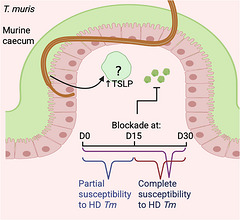

## 1 Introduction

Thymic stromal lymphopoietin (TSLP) is a four‐alpha helix bundle cytokine, emerging early in vertebrate evolution as a paralog of lymphocyte growth factor IL‐7 [1], with orthologs in both humans and mice. A key cytokine in promoting the type two immune response against the intestinal helminth *Trichuris muris* (*Tm*), whipworm [2], TSLP has been a molecule of substantial interest in the field of human atopy since first observed to produce a model of murine asthma when overexpressed in the lung [3], with TSLP‐neutralising tezepelumab having recently shown promising phase III clinical trial results in the treatment of severe asthma [4, 5]. Despite this, key aspects of TSLP's biology remain unclear: despite being broadly characterised as an epithelial alarmin [6, 7, 8, 9, 10], recent work in mice has indicated murine *Tslp* expression to be fibroblast‐restricted in the small intestine [11] and macrophage‐restricted in the skin [12] during challenge (feeding and *Leishmania* infection, respectively). Additionally, both papers characterise the alarmin as acting directly on type two innate lymphoid cells to enable their function, in contrast to research suggesting TSLP action on dendritic cells [13, 14]. Although the level of functional conservation between murine and human TSLP is not clear in light of the short inhibitory form of TSLP expressed by humans [15] but seemingly not by mice, human lung macrophage production of TSLP in response to stimulus ex vivo has been observed [16], suggesting that nonepithelial expression of TSLP in various murine tissues may correspond to unstudied TSLP sources in humans. Additionally, the effectiveness of tezepelumab in treating human asthma, in which adaptive immune responses to allergens would be overwhelmingly long established, would support TSLP as primarily functioning to enable lymphoid effector cell production of pro‐asthmatic cytokines, rather than acting on antigen presenting cells.

*Tm* is an orally transmitted, caecum‐dwelling gastrointestinal helminth and a closely related species to the widespread human‐infecting *Trichuris trichiura*. While low‐level infection results in type one adaptive immune responses and chronic infection, high‐level infections (high doses of eggs, HD) result in type two adaptive immunity and IL‐13‐mediated epithelial expulsion of the parasite from the caecum [17, 18]. HD *Tm infection* is therefore a useful model of the necessary conditions for the induction of type two immunity. Among helminths, TSLP appears uniquely necessary for supporting type two immune responses in this model [14]. This makes HD *Tm* infection an ideal candidate for studying natural induction of TSLP, and examination of how TSLP contributes to a functional immune response leading to parasite expulsion.

Here, using conditional knockout models, we demonstrate that TSLP exerts its impact on immunity predominantly during the third and fourth weeks post‐infection, consistent with publications from other groups suggesting a role for TSLP in licensing effector cell function in tissues. We additionally find caecal epithelial cells to be poor sources of *Tslp* both during homeostasis and HD *Tm* infection, and that conditional knockout from caecal epithelium, telocytes, or CCL24+ cells does not impact immunity to HD *Tm* infection.

## 2 Results

### 2.1 TSLP Contributes to HD *Trichuris* Expulsion Primarily From Day 14 Post‐Infection Onwards

**FIGURE 1:**
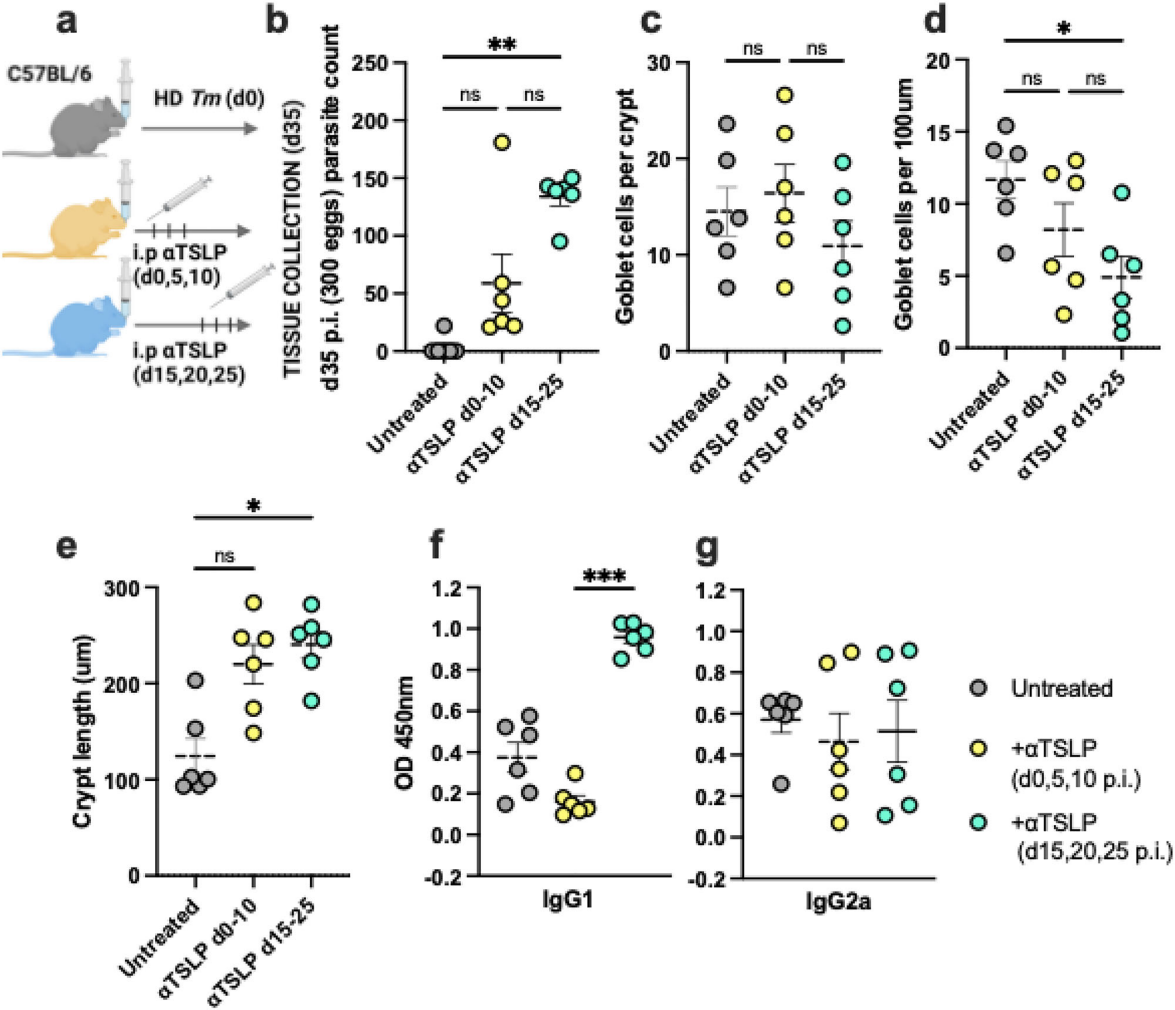
Impact of early and late TSLP neutralisation on parasite expulsion, antibody responses, and stromal immunity following HD *Tm* infection. (a, b) C57BL/6 mice were infected with 400 *Tm* eggs and treated with 100 µg of anti‐TSLP antibody intraperitoneally in two different dosage regimes, as well as an untreated group, harvested at day 35 p.i. (a) Experimental summary. (b) Counts of adult parasites recovered from caeca in each group (Kruskal–Wallace testing). The number of goblet cells per crypt (b), goblet cells per 100 µL of crypt (d), and length of crypts (e) were quantified for all groups via analysis of Alcian blue histological staining in ImageJ (ordinary one‐way ANOVA testing). Serum level of parasite‐specific IgG1a (f) and IgG2a/c (g) was measured via ELISA and graphed in arbitrary optical density units (Kruskal–Wallis testing). Each point in (c–e) used in (f–h) represents an average of at least six crypts from one mouse. *n* = 6 mice per group, between 12 and 16 weeks old at the time of harvest. *p < 0.05, **p < 0.01, ***p < 0.005, *p* < 0.05 are not displayed.

In the context of previous publications indicating a role for TSLP in lymphoid effector cell licensing rather than antigen presenting cell polarisation, a timed neutralisation experiment was performed in which one group of mice received anti‐TSLP antibody treatment at days 0, 5, and 10 (early) p.i. with a high dose of *Trichuris muris* eggs (HD *Tm*), administered orally, while another group was treated at days 15, 20, and 25 (late) p.i., alongside an infected but untreated group (Figure 1a). Although the early anti‐TSLP‐treated group retained parasites at d35 p.i., this was not significantly different from the untreated mice, which had almost entirely expelled their parasites by this time point. However, late anti‐TSLP‐treated mice retained many more worms (>twofold higher parasite burden), exhibiting a significant increase over untreated mice (Figure 1b). This was accompanied by a decreased goblet cell count per micron of crypt (Figure 1c,d) and an increased crypt length (Figure 1e) in late‐treated mice versus untreated controls, indicative of impaired goblet cell hyperplasia and chronic infection, respectively. In these epithelial readouts, the early‐treated group exhibited nonsignificant changes intermediate between untreated and late‐treated mice, potentially suggestive of the administered antibody persisting into late infection. Unusually, late‐treated mice also showed increased parasite‐specific IgG1 in serum (Figure 1f), normally associated with TH2 immunity and parasite expulsion. IgG2a levels, associated with chronic infection, were largely unimpacted in both groups (Figure 1g). Overall, TSLP appears to primarily contribute to immunity during the expulsion phase of infection.

### 2.2 *Tslp* is Poorly Expressed by the Caecal Epithelial Compartment and Not Substantially Upregulated in the Caecum During HD *Trichuris* Infection

**FIGURE 2:**
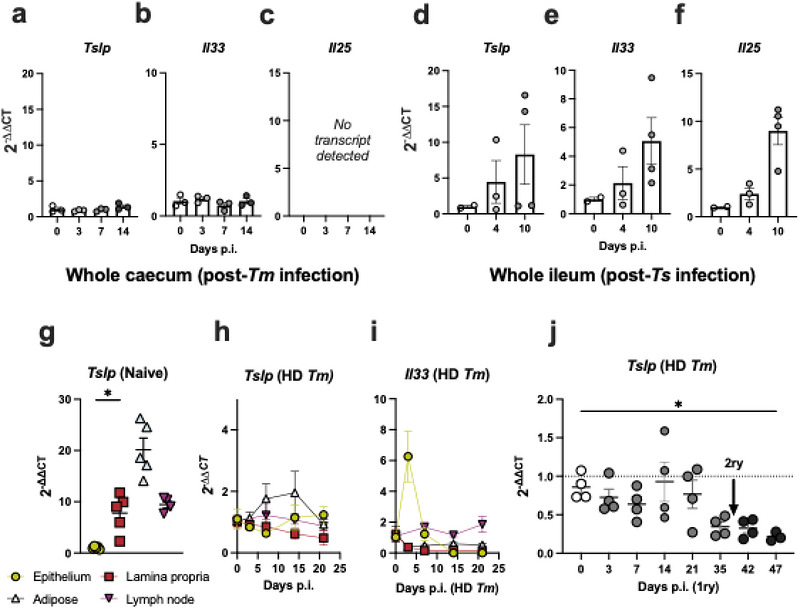
*Tslp* expression within caecal compartments and associated tissues, and tissue alarmin expression during *Trichinella* and *Trichuris* infection. (a–c) Whole caecal intestinal tissue harvested from 300‐egg dose *Trichuris muris (Tm)*‐infected mice at the listed p.i. was examined for alarmin transcription via qPCR, targeting *Tslp* (a), *Il33* (b), and *Il25* (c) transcripts. (d‐f) Whole ileal tissue from *Trichinella spiralis*‐infected mice was similarly examined at different timepoints (Kruskal–Wallis, *n* = 2–4 mice per group). (g) The epithelial compartment was isolated from the caeca of naïve mice and harvested alongside the remaining lamina propria tissue, caecum‐adjacent adipose tissue, and the caecum‐draining lymph node: qPCR was performed on these samples targeting *Tslp* and *Rpl13*, and the fold‐change over epithelial ΔCT calculated for all samples (Kruskal–Wallis). (h, i) The procedure described in (g) was repeated for samples from mice infected with 300 *Tm* eggs harvested at days 0, 3, 7, 14, and 21 p.i., with ΔΔCT values instead calculated against day 0 results for each tissue, for both *Tslp* (h) and *Il33* (i) (Mixed effects testing, *n* = 3–5 per group). (j) Whole caecal tissue was harvested as in (a–c), with the addition of a secondary 300‐egg infection administered on day 39 post‐primary infection, with qPCR performed targeting *Tslp* (n = 3‐4 per group). All samples were generated from male C57BL/6 mice between 8 and 16 weeks old. * *p* < 0.05; *p* > 0.05 are not displayed.

To examine any changes in expression of *Tslp* by epithelial cells during HD *Tm* infection, which has not, to our knowledge, been demonstrated in C57BL/6 mice, qPCR was performed firstly on whole caecum samples from C57BL/6 mice at days 0, 3, 7, and 14 p.i. with HD *Tm*, analysing transcription of *Tslp*, *Il33*, and *Il25*. *Tslp* and *Il33* exhibited no trending increase at any time point over naïve samples (Figure 2a,b) while *Il25* transcript was undetected in all caecal samples (Figure 2c), consistent with poor tuft cell counts in the caecum compared with the small intestine observed previously [19]. As a positive control, whole small intestinal samples from *Trichinella*‐infected mice were similarly analysed, and trends towards increased transcription of all alarmins were apparent at d10 p.i. (Figure 2d–f). Next, naïve expression of *Tslp* across caecal compartments and associated tissues was quantified (Figure 2g): adipose tissue has been recently observed to produce *Tslp* during *H. polygyrus bakeri* infection [20], and here exhibited the highest naïve expression level of *Tslp* among tissues sampled. Flow cytometry analysis indicated expansion of adipose stromal cells during HD *Tm* infection, resembling that observed by Kabat et al. following *H. polygyrus bakeri* infection [20]; however, qPCR data from caecum‐associated adipose and ELISA of isolated mesenchymal adipose tissue progenitor cells indicated no significant increase in *Tslp* expression or protein production from adipose tissue at d14 p.i. (Figure S1). The epithelial samples contained, on average, approximately eightfold lower levels of *Tslp* transcript than the lamina propria samples (also containing the submucosa and muscularis layers), 10‐fold lower than lymph node samples, and 20‐fold lower than adipose tissue. Examining these compartments/tissues separately across the course of HD *Tm* infection, no significant increase in *Tslp* transcription was observed in any tissue (Figure 2h), while *Il33* exhibited a trend towards upregulation early in the epithelial compartment (Figure 2i). No *Il25* was detected in any samples during this experiment (data not shown). In light of our data suggesting important TSLP functionality at later stages of infection, *Tslp* transcription was measured in whole caecal samples across a longer time course, in which a secondary infection was also administered at day 39 post‐primary infection (Figure 2j). Again, no indication of significant upregulation was detected, and indeed *Tslp* expression decreased significantly at day 47 p.i., possibly owing to tissue composition changes such as expansion of the caecal patch and increased epithelial cell proportion. Taken together, these data suggest against a robust transcriptional alarmin response in the caecum and associated tissues during HD *Tm*, despite its clear importance to parasite expulsion.

### 2.3 Caecal Epithelial Knockout of TSLP does not Replicate the Susceptibility to High‐Dose *Trichuris* Infection Produced by Systemic TSLP Neutralisation

**FIGURE 3:**
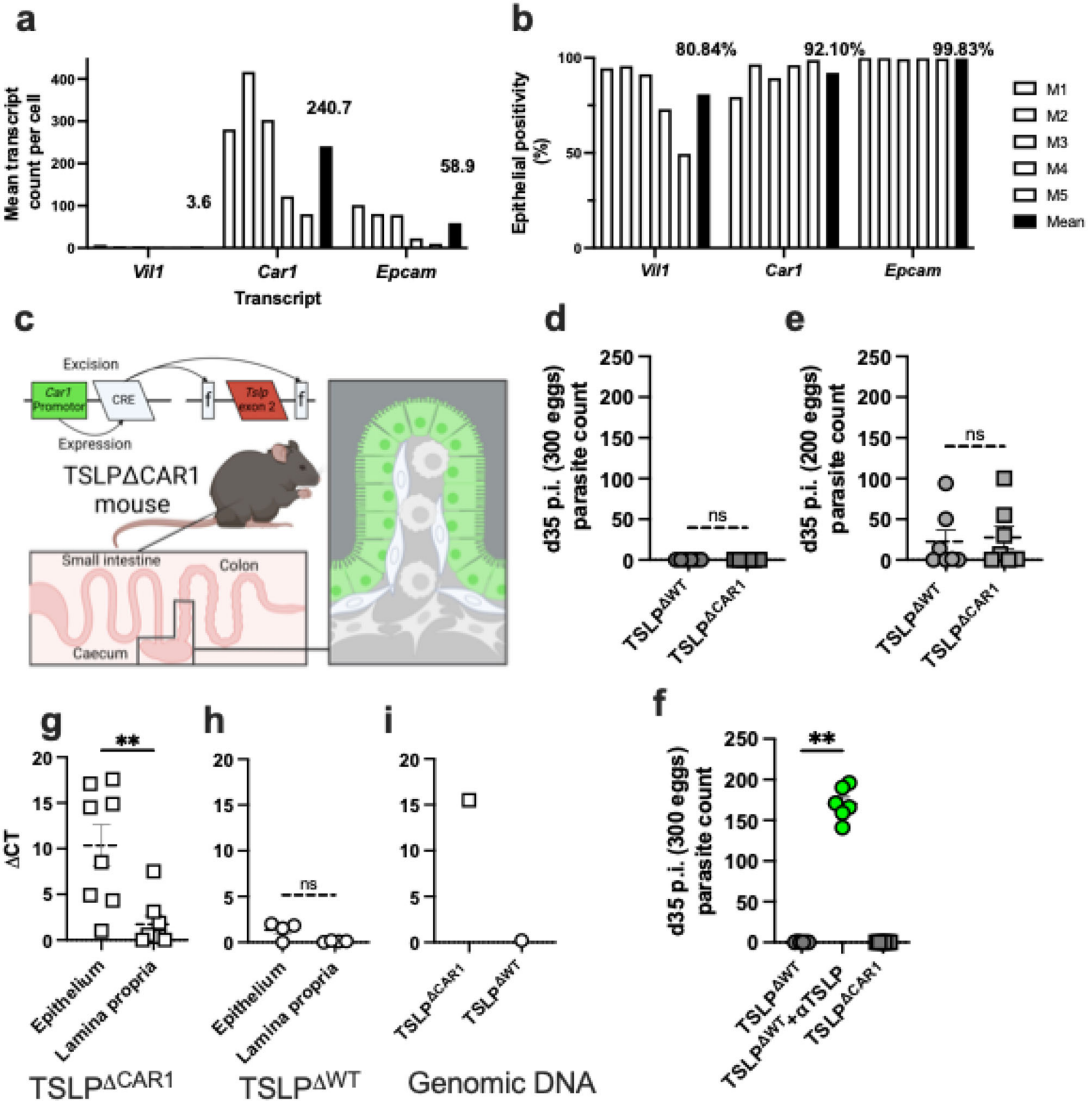
Validation and HD *Tm* susceptibility of the TSLPΔCAR1 caecal epithelial TSLP knockout mouse.(a, b) Single‐cell sequencing data from epithelial cells of caeca from five female C57BL/6 mice at 8 to 18 weeks old, produced as described here, with underlying matrix data deposited at GSE168448 in the NCBI GEO repository, were analysed. Expression data graphed as a percentage of positive cells (>0 transcripts) is given in (a), while average transcript expression is given in (b) (Kruskal–Wallis testing). Mean bars are value labelled for each transcript. (c) An overview of the TSLPΔCAR1 mouse, knocking out *Tslp* expression in caecal and proximal colonic epithelial cells. Counts of adult parasites recovered from caeca in TSLPΔCAR1 and littermate control mice (TSLPΔWT) are shown, following 300‐egg (d) and 200‐egg (e) infections, at day 35 p.i. with *Tm* (Mann–Whitney testing, *n* = 6–7 mice per group). (f) Groups and infection as in (d) repeated, plus a group of littermate control mice administered 100µg of anti‐TSLP antibody via intraperitoneal injection every five days from d0 to d25 p.i. (Kruskal–Wallis testing, *n* = 6 mice per group). *Cre* recombinase transcript ΔCT values generated against *Rpl13* housekeeping transcript, detected via RT‐qPCR results from cDNA reverse transcribed from RNA isolated from enzymatically separated epithelial cells and remaining lamina propria (plus submucosa and muscularis layers), given for naïve TSLPΔCAR1 mice (e) and littermate controls (h) (Wilcoxon testing, *n* = 7 mice per group). The same reaction was performed on genomic DNA from both groups in (i) (*n* = 1 mouse per group). Mice between 12 and 16 weeks old at the time of harvest. * *p* < 0.05, ** *p* < 0.01, *p* > 0.05 are not displayed.

Following the absence of *Tslp* upregulation during HD *Tm* infection and the poor expression by caecal epithelial cells, we opted to produce a conditional knockout mouse to remove expression of TSLP in caecal epithelial cells, and examine any impairment of the immune response to HD *Tm* infection. Observations of the Villin^β‐gal^ mouse from our lab previously suggested relatively poor expression of Villin in the caecum (data not shown), commonly used in Cre‐flox systems for producing epithelial cell knockout in the small intestine [21]. To verify this, we examined a publicly available single‐cell sequencing dataset of the murine caecal epithelium for expression of *Vil1*, *Car1*, as well as *Epcam* [22]. In terms of both mean transcript count per epithelial cell as well as percentage of positive cells, *Car1* exhibited higher expression in caecal epithelium than *Vil1*, and was thus selected as the more suitable Cre promoter site (Figure 3a,b). A Car1‐Cre TSLP(flox/flox) mouse was created, in which exon 2 of the *Tslp* gene is flanked by LoxP sequences, and Cre recombinase enzyme is expressed under the *Car1* promoter, constitutively expressed by proximal colonic and caecal epithelial cells, causing excision in *Car1*+ cells of genomic DNA encoding TSLP exon 2, rendering functional TSLP protein untranscribable in this cell type (Figure 3c). Although *Car1* is expressed by mast cells in addition to epithelial cells [23], mast cells appear to play little role in the expulsion of HD *Tm* [24], limiting any caveats regarding off‐target effects. TSLPΔCAR1 mice, as well as Cre‐negative littermates (TSLPΔWT), were infected with 300‐ or 200‐egg doses and adult parasites in the caecum counted at day 35 p.i. (Figure 3d,e). A 200‐egg dose was included as a more sensitive model of resistance, given the correlation between higher dose and stronger type two immunity. No differences were observed in expulsion between TSLPΔCAR1 and TSLPΔWT mice in either experiment, with complete expulsion of the 300‐egg dose, and equivalent partial expulsion of the 200‐egg dose between groups. To verify the previously reported requirement for TSLP signalling in expulsion of HD *Tm* infection [2, 14], a 300‐egg infection was repeated with the addition of a group of littermates treated with anti‐TSLP antibody every 5 days throughout infection, starting at day 0. In this group, all mice remained infected by d35 p.i., while untreated TSLPΔCAR1 mice and wild‐type littermates expelled all parasites (Figure 3f). To validate Cre recombinase expression in the TSLPΔCAR1 mouse, epithelial cells of the caecum were isolated from the caeca of naïve TSLPΔCAR1 mice and littermates, and qPCR performed to measure Cre recombinase RNA expression: Cre recombinase RNA expression was observed in TSLPΔCAR1 epithelial samples to be >100‐fold higher (approximately eight cycles faster to detection) than in the lamina propria sample, or in either sample from TSLPΔWT mice (Figure 3g,h). Expression level in TSLPΔCAR1 epithelial samples, measured by ΔCT versus *Rpl13*, was in most samples comparable to that obtained from TSLPΔCAR1 genomic DNA from ear punches (Figure 3i), suggesting strong expression of the enzyme in the caecal epithelium in this knockout. Together, these data indicate no role for caecal epithelium‐derived *Tslp* production in contributing to expulsion of HD *Tm*.

### 2.4 TSLP Neutralisation, but not Epithelial TSLP Knockout of TSLP, Produces Impaired Epithelial Responses to HD *Trichuris* Infection

**FIGURE 4:**
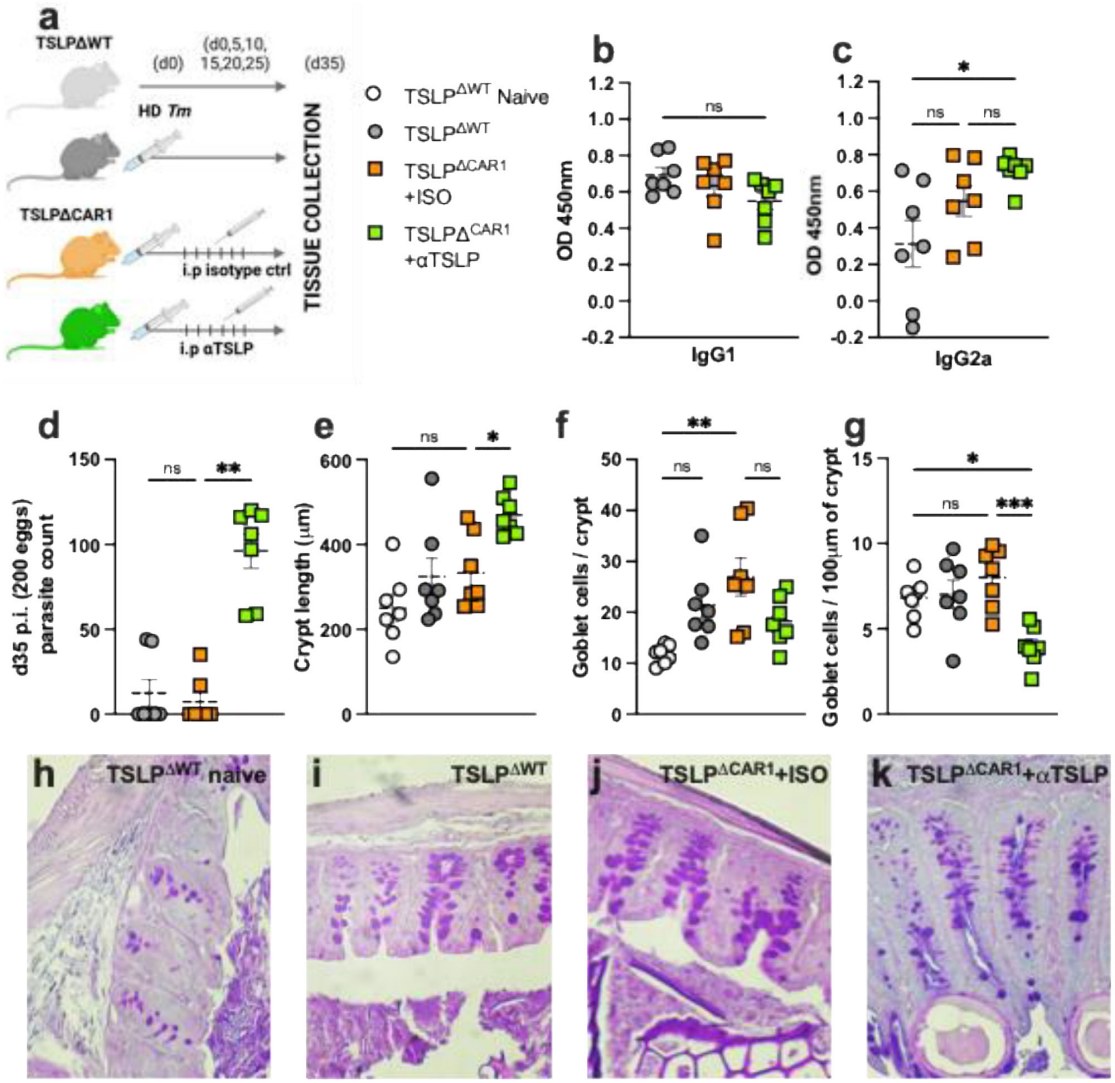
Immune readouts during *Trichuris* infection in epithelial conditional TSLP knockout and systemically TSLP neutralised mice. (a) Experiment overview: TSLPΔCAR1 mice and littermate controls were infected with 200 *Tm* eggs, TSLPΔCAR1 mice being separated into an isotype control‐treated (rat IgG2a) group and an anti‐TSLP (rat IgG2a) treated group, each receiving 100 µg of antibody administered intraperitoneally every 5 days from d0–25 p.i., before sample collection at d35 p.i. (b) Counts of adult parasites recovered from the caeca in each group. Serum level of parasite‐specific IgG1a (c) and IgG2a/c (d), measured via ELISA at 1 in 20 dilution and graphed in arbitrary optical density units, for infected groups (a–c, Kruskal–Wallis testing). Length of caecal crypts (e), number of goblet cells per crypt (f), and goblet cells per 100 µL of crypt (g) were quantified for all groups via analysis of H&E histological staining in ImageJ (ordinary one‐way ANOVA). (h–k) Representative images of H&E sections from each group used in quantification. Each point used in (d–f) represents an average of at least six crypts from one mouse. *n* = 7 mice per group, between 12 and 16 weeks old at the time of harvest. * *p* < 0.05, ** *p* < 0.01, *** *p* < 0.005.

To gain insight into how immunity to HD *Tm* infection might be altered by impaired TSLP signalling, levels of parasite‐specific antibody in serum and changes to the epithelial layer of the caecum were examined under several conditions: wild‐type littermates were separated into uninfected (TSLPΔWT naive) and day 35 p.i. (TSLPΔWT) groups, while epithelial TSLP knockouts were separated into groups treated with either isotype control antibody (TSLPΔCAR1+ISO, or “isotype treated”) or anti‐TSLP antibody (TSLPΔCAR1+αTSLP, or “αTSLP treated”) every 5 days from days 0 to 25, before harvest at day 35 (Figure 4a). Serum parasite‐specific IgG1, used as a proxy of TH2 differentiation in the lymph node [25], was unimpacted by either epithelial TSLP knockout or TSLP neutralisation (Figure 4b). TH1‐associated IgG2a was likewise statistically not significant between αTSLP‐treated and isotype‐treated epithelial knockouts, despite an increase in the αTSLP‐treated group over untreated wild‐type mice, in line with previously published data from TSLPR knockout mice [2] (Figure 4c), suggesting only a marginal impact of TSLP neutralisation on B cell responses. Partial to total expulsion of parasites was observed in untreated littermates and isotype‐treated epithelial knockouts, in contrast to the chronically infected αTSLP‐treated mice (Figure 4d). Examining epithelial responses associated with IL‐13‐mediated parasite expulsion using H&E staining of tissue sections from the caecum, increased crypt length relative to naïve WT samples, indicative of chronic infection, was only observed in the αTSLP‐treated mice (Figure 4e), which likewise differed from infected wild‐type mice in having a reduced goblet cell count when normalised for changes to crypt length (Figure 4f,g). Isotype‐treated epithelial knockouts did not differ from either naïve or infected littermates in crypt length or indications of goblet cell hyperplasia, indicating no impairment of worm expulsion (representative images given in Figure 4h–k). These data indicate that while epithelial TSLP does not contribute to HD *Tm* immunity, TSLP is necessary for epithelial changes in the caecum as well as parasite expulsion, but appears to have little impact on B cell responses.

### 2.5 In Situ Hybridisation Indicates Non‐Epithelial, Non‐Telocyte Upregulation of *Tslp* in the Caecum During HD *Trichuris* Infection

**FIGURE 5:**
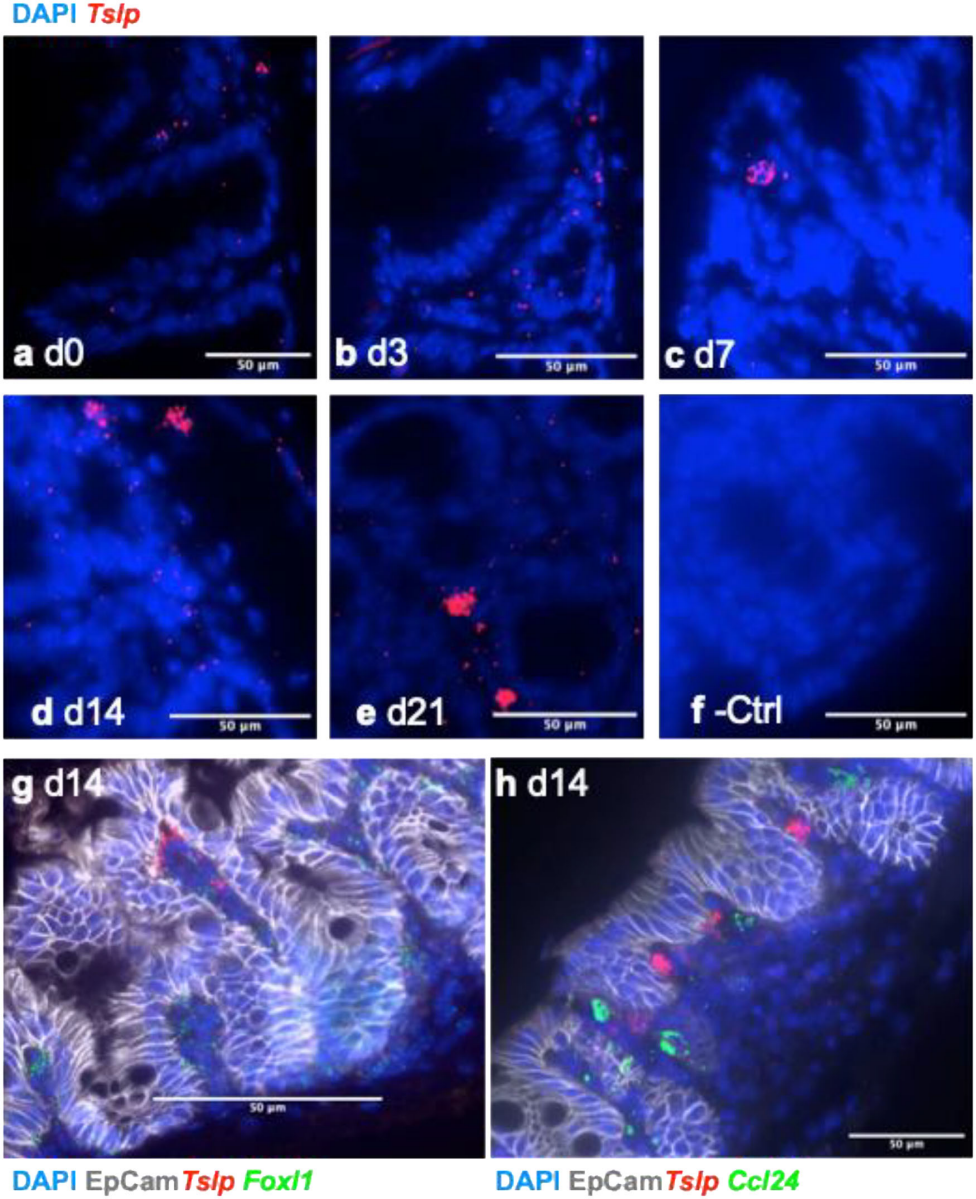
*Tslp* transcript imaging and quantification in the HD Tm‐infected caecum. Representative images of RNAscope‐stained FFPE sections from male C57BL/6 mouse caeca post‐HD *Tm* (300‐eggs) infection in naïve mice (a), and mice at days 3 (b), 7 (c), 14 (d), and 21 (e) post‐infection. *Tslp* transcript (in red) counterstained with DAPI for nuclei (blue) in sections represented by (a–e), with a random oligomer probe substituted for *Tslp* in negative control sections (f). (g, h) Day 14 p.i. samples were used to generate sections stained similarly, with the addition of EpCAM antibody co‐staining, shown in grey, and either Foxl1 (g) or Ccl24 (h) RNAscope probes, shown in green. Mice were between 12 and 16 weeks old at the time of harvest.

RNAscope was used to visualise expression of *Tslp* mRNA in the caecum throughout HD *Tm* infection. *Tslp*+ cells were detected at all timepoints, including naïve mice (Figure 5a), localised to rare lamina propria‐ and submucosa‐resident cells (Figure 5b–e). Expression in epithelial cells was negligible, with cells directly in contact with the intestinal lumen rarely exhibiting more than 1 transcript, and the strongest expression was observed during days 14 and 21 p.i. sections, supporting the idea that Tslp exerts influence on immunity during later timepoints of infection. Additionally, considering recent data indicating Foxl1+ telocytes as potential contributors to *Tslp* production in the small intestine and CCL24+ cells as *Tslp* producers in the skin during *Leishmania* infection, RNAscope probes for these two transcripts were also used alongside *Tslp* and EpCAM on day 14 samples (Figure 5g,h). As expected, the *Tslp* transcript above the negative control random probe levels was absent in EpCAM+ cells. *Ccl24* appeared to be induced during infection in cells distinct from those upregulating *Tslp*, while *Tslp* was produced in both *Foxl1*+ and *Foxl1*‐ cells at this time point. To determine any impact of *Tslp* deletion in these cell types on the outcome of HD *Tm* infection, additional conditional knockouts were created targeting *Ccl24* and *Foxl1* expression of *Tslp*, with worm burdens and epithelial changes examined as in Figure 4. Neither knockout displayed any alterations in worm burden following 200‐ or 300‐egg infection, nor were there alterations to goblet cell hyperplasia (Figures S2 and S3), confirming neither cell type as being key TSLP producers during HD *Tm* immunity. Together, these imaging experiments indicate a caecal TSLP‐producing cell in the caecum, distinct from TSLP expressors characterised in other tissues and challenges, directly or indirectly influencing TH2 responses during the expulsion phase of immunity to HD *Tm* infection.

## 3 Discussion

TSLP's contribution to HD *Tm* immunity being primarily during late infection, when interaction with effector lymphoid cells appears more likely than antigen presenting cell polarisation, is in part surprising but in accordance with recent examination of TSLPs immunological function in other tissues and contexts: while previous studies have posited dendritic cell detection of epithelium‐produced TSLP as the key TSLP contribution to HD *Tm* immunity [13], various papers have recently documented TSLPR expression on ILC2s and TH2 cells [12, 20, 26, 27, 28, 29, 30, 31] to a far greater extent than has so far been demonstrated for dendritic cell detection of TSLP in vivo. The epithelial alarmin framework has been increasingly eroded, as is the conception of alarmin action on antigen‐presenting cells, and considering the contribution of alarmins to atopic diseases, they suffer from a noticeable lack of mechanistic characterisation. *Tm*’s TSLP‐dependent expulsion model presents an ideal system: unlike some small intestinal parasite infections, HD *Tm* appears to be strongly reliant on TH2 responses [32] with only a small contribution from ILC2s to parasite clearance [33], which, alongside the demonstrated role for TSLP in licensing TH2 activity, is consistent with a role for TSLP during the expulsion phase of HD *Tm* infection. The absence of a broad transcriptional alarmin response and poor epithelial expression of *Tslp* both in naïve and during HD *Tm* infection has not been previously published in detail, but is consistent with current work: TSLP expression in the caecal epithelial layer in C57BL/6 mice has been observed via antibody staining [2], but recent single cell sequencing examination of the caecum in early infection with HD *Tm* (days 1 and 3 p.i.) uncovered no changes in *Tslp* expression, and minimal expression in IECs [34], and no other published data to our knowledge supports epithelial upregulation of *Tslp* in C57BL/6 mice. This is demonstrated concretely *via* the Car1‐Cre conditional knockout, for which even 200‐egg doses produced partial susceptibility, and no effect was observed on either goblet cell responses or parasite expulsion in epithelial knockout mice. This is in stark contrast to the robust loss of immunity in anti‐TSLP‐treated mice, as documented in previous work. The decrease in goblet cell numbers per crypt below even naïve levels in infected and anti‐TSLP‐treated mice suggests that homeostatic TSLP may support homeostatic ILC2 IL‐13 production and thus promote goblet cell differentiation. The minor effects of TSLP blockade on parasite‐specific antibody responses also support a role for TSLP in licensing TH2 cells in tissue rather than shaping adaptive immunity in the lymph node itself. However, recent interesting work has noted a role for TSLP in enabling T‐B cell crosstalk [35]. Other recent work examining follicular helper T cell responses suggests a broadly pro‐TH1 signature in these cells following *Tm* infection, in contrast to *Hp* infection [36], in consensus with previous work indicating a limited role for B cell‐produced antibodies in directly shaping protective immunity to HD *Tm* infection [24, 37]. As a result, the effects of anti‐TSLP treatment of antibody responses may be mechanistically distinct from the susceptibility to HD *Tm* infection that it also produces. The absence of notable upregulation of *Tslp* observed by qPCR, taken with RNAscope data indicating clear *Tslp* transcription increases in the lamina propria during infection, suggests caecal *Tslp* responses to be highly localised, upregulated only in crypts contacting parasites, diluting any observable increases during analysis of whole tissue. *Foxl1*+ telocytes, a subepithelial fibroblast subset recently indicated to be *Tslp* producers in the small intestine during feeding, appeared here to potentially contribute to *Tslp* production, but conditional knockout indicated no key role in immunity. The aforementioned study includes reporter mouse imaging and flow cytometric data strongly indicating lamina propria‐resident cells as *Tslp* producers during HD *Tm*, and demonstrates impaired ILC2 production of key expulsion‐linked cytokine IL‐13 when TSLP expression is deleted from stromal cells. The study does not, however, examine functional effects on parasite expulsion, and considering the relatively minor impact of ILC2 deletion on expulsion of HD *Tm* infection [33], additional research is required to characterise both the key sources and recipients during infection. Alterations in ILC2 cytokine production being demonstrated in the large intestine, rather than the caecum, whether stromal cells represent the key TSLP source during HD *Tm* immunity, also creates space for additional work utilising new spatial proteomic and transcriptomic approaches for examining whether the caecum or large intestine represents the key site for TSLP production and resulting immunity. The dynamics and importance of TSLP‐TH2 cell interactions during infection also remain to be determined: pilot single‐cell sequencing data from our lab indicate more robust expression of the TSLPR by TH2 cells than by ILC2s in the caecum during infection (data not shown), but further conditional knockout and *in vitro* experiments would produce interesting results in this direction. *Ccl24*+ macrophages, demonstrated as key TSLP producers in the skin during *Leishmaniasis* [12], were observed to strongly transcribe *Ccl24* during HD *Tm* infection, but were distinctly separate from *Tslp*+ cells, were likewise confirmed here as not being key *Tslp* producers using conditional knockout experiments. A recent study has linked stromal TSLP production to ILC2 activation and indicated upregulation during HD *Tm* infection: no characterisation of either ILC2 detection or stromal production of TSLP's impact on functional parasite immunity was presented, but our results here are suggestive of a role for non‐telocyte stromal TSLP production as being potentially supportive of functional immunity, or that redundancy exists between *Tslp*‐producing cell populations.

Here, the concept of TSLP responses in the intestine as being epithelial and as contributing to immunity early on in infection before the expulsion phase is not supported, and creates space in the field for characterisation of a new mechanism by which TSLP influences the immune system, with important implications for diseases at mucosal sites in which TSLP plays an important role, such as allergic asthma. The level of functional conservation between murine and human TSLP functionality is not clear, considering the short inhibitory form of TSLP expressed by humans [15] but seemingly not by mice. Recent single‐cell sequencing data from the human lung observed expression of *Il33* by epithelial cells, but not *Tslp* [38]. Another study of human lung samples observed a correlation between epithelium expression of EGR1, Il33 expression, and asthma, while no correlation between EGR1 and *Tslp* was observed in epithelial cells. Human lung macrophage production of TSLP in response to stimulus *ex vivo* has been observed [16]; however, suggestive that recently observed non‐epithelial expression of TSLP in various murine tissues may shed light on unstudied TSLP sources in humans. Significant gaps remain in the field regarding the specific mechanism by which TSLP, among other alarmins, contributes to the immunology of asthma: detailed investigation along the lines of inquiry followed here would be highly informative in this area of study.

## 4 Methods

### 4.1 Mice

C57BL/6J.*Tslp*Em1Uman mice (TSLP‐floxed, or TSLP^f/f^) mice were generated by the University of Manchester Genome Editing Unit, the two loxP sites being integrated in the intergenic region upstream (5’) of the gene and within the *Tslp* intron between exons 2 and 3 (3’), using a CRISPR‐Cas9 system to integrate long single stranded DNA, generated as described by [39], administered via pronuclear microinjection of cryopreserved 1‐cell embryos alongside an sgRNA (Sigma‐Aldrich, g295 – gaggctctccccgcttagag, g330 – ttttacatgccaaatgtgtg), which were implanted into surrogate psuedopregnant mice after overnight culture of the embryos. Genotyping was performed with REDExtract‐N‐Amp Tissue PCR Kit (Sigma‐Aldrich) to validate insertions via gDNA ear punches in weaned mice. C57BL/6 mice were purchased from Charles River or bred in‐house at the University of Manchester Biological Services Unit (derived initially from Envigo C57BL/6J). Foxl1‐Cre (officially B6;SJL‐Tg (Foxl1‐cre)1Khk/J) and Car1‐Cre (officially C57BL/6‐Tg(Car1‐cre)5Flt/J) transgenic mice were both purchased from the Jackson Laboratory, and separately crossed with homozygous TSLP‐floxed mice to produce TSLPΔTELO (Foxl1(Cre)Tslp(flox/flox)) and TSLPΔCAR1 (Car1(Cre)Tslp(flox/flox)) mice, respectively, alongside wild‐type littermate controls (Foxl1/Car1(WT)Tslp(flox/flox)). TSLPΔCCL24 (Ccl24(Cre)Tslp(flox/flox)) mice were donated by Dr David Sacks (NIAID, NIH, Bethesda), produced as described in the study by Lee et al. [12]. All mice were maintained in a specific pathogen‐free (SPF) facility at the University of Manchester, in a 12:12 h light:dark cycle at 21 ± 5°C and 55 ± 10% humidity. Mice were housed in cages of one to six, with rodent chow and sterile water provided ad libitum. All mice were euthanised via rising concentration of CO_2_. Experiments were performed in accordance with the United Kingdom Animals (Scientific Procedures) Act of 1986, under project licenses P043A082 (2021‐2023) and PP0172300 (2024‐2025) and were subject to local ethical review by the University of Manchester Animal Welfare and Ethical Review Body (AWERB) and followed ARRIVE 2.0 guidelines. The mice utilised in these experiments were not randomised, but cages were randomly assigned to different treatment groups for the experiments. All mice used for experiments were between 8 and 16 weeks of age. Knockout mouse experiments made use of both sexes of mice, while C57BL/6 experiments used male mice only. Details of the ages and sexes of mice used for each experiment are provided in the figure legends.

### 4.2 Trichuris muris

The Edinburgh strain of *T. muris* was used in all experiments, originally obtained from The Wellcome Research Laboratories, London, and is routinely passaged within our laboratory. For infections, mice were administered between 200 and 400 eggs via oral gavage in Milli‐Q water, using eggs between 60% and 75% infectivity, as measured via batch test infection of SCID immunodeficient mice, bred in‐house at the University of Manchester (strain originally obtained from the Fox Chase Cancer Centre, Philadelphia). For parasite count data, infected mice were euthanised, and the caeca and proximal colon were harvested. Tissue was opened longitudinally, and contents washed out with water, before counting of adult parasites by eye using the MZ75 dissection microscope (Leica). For E/S acquisition, X‐linked severe combined immunodeficient (SCID) mice were infected with 200 *T. muris* eggs by oral gavage, and their caeca were harvested at day 42 p.i. into RPMI‐1640 (Sigma‐Aldrich) plus 500 U/mL penicillin and 500 µg/mL streptomycin (Sigma Aldrich) (RPMI+P/S). Mice were euthanised, and caeca stored in pre‐warmed (approx. 40°C) RPMI were extracted from the caecum using fine forceps and incubated in RPMI+P/S for 4 h at 37°C, with the media being harvested and replaced for a second 16 h incubation. Both lots of media were centrifuged in 50 mL tubes (Falcon) to pellet eggs, and the supernatant was collected into a new 50 mL tube for E/S production. Pelleted eggs were resuspended in Milli‐Q (Sigma‐Aldrich) water, passed through a 70 µm filter to remove adult parasites and intestinal debris, and embryonated via incubation in T75 culture flasks (Corning) for 6–8 weeks. Embryonated eggs were transferred to 4°C storage for use in infecting mice, after infectivity testing to ensure >50% infectivity (i.e., 200 egg administration to a mouse producing >100 embedded larvae at a time point before expulsion, d12‐16 p.i.). The supernatant separated from eggs for E/S production was first filtered through a 0.22 µm syringe filter (Sartorius). Concentration was performed using Amicon Ultra centrifuge units, and the resulting media was centrifuged at 3000×*g* for 15 min at 4°C. The resulting media was dialysed against PBS (pH 7.4, Gibco) for 24 h at 4°C, then the protein concentration of the E/S was determined using a Nanodrop (NanoDrop One, Thermo Fisher). Resulting E/S was aliquoted and stored at −20°C until use.

### 4.3 In Vivo Administration of Antibodies

Treated mice were injected intraperitoneally with 100 µL of 1 mg/mL rat‐anti‐mouse anti‐TSLP (Amgen, IgG2a not commercially available) every 5 days on alternating sides. Isotype control‐treated mice received 100 µL of isotype control antibody (BioXcell, cat#BE0085) at the same timepoints.

### 4.4 Anti‐Parasite Immunoglobulin ELISA

Flat‐bottomed 96‐well plates (Corning) were coated with 5 µg/mL of parasite E/S diluted in sodium carbonate bicarbonate buffer (pH 9.6) and incubated overnight at 4°C. The following day, plates were washed five times (all wash steps were performed with 200 µL per well of PBS plus 0.05% Tween‐20 (Sigma)), using the AquaMax 4000 (Molecular Devices), and incubated with 100 µL of 3% BSA (Sigma) in PBS for 1 h at room temperature (RT). Block was tipped from plates, and 100 µL of serum dilution (top row 1:20, diluted 1:3, serially) was added to each well, and incubated for 90 min at RT. Plates were washed three times and incubated with biotin‐conjugated αIgG1 (cat#MCA336B, BioRad) or αIgG2a (cat#553388, BD Biosciences) at 1:1000 in PBS for 1 h at RT. Plates were washed and incubated with 1:1000 streptavidin‐POD conjugate (Roche) for 1 h at RT. Plates were washed and incubated with a 1:1 solution of development substrates A and B from the BD OptEIA TMB substrate reagent set (BD Biosciences). Wells were allowed to develop before a stop solution was added (50 µL of 2 M sulphuric acid). Plates were read at 405 nm using a VersaMax plate reader, and after verification of normal dilution:OD relationships for samples, a concentration of 1:120 was graphed for each sample on the plates.

### 4.5 Histological Staining and Quantification

Caecal tip tissue dissected from mice was fixed by immersion in 4% neutral buffered formalin for 16 h, followed by transfer into 70% ethanol (Fisher) and wax embedding. Sections were cut at 5 µm thickness via microtome (Leica RM2255) and mounted onto Superfrost Plus slides (Epredia). Haematoxylin and eosin (H&E) staining was performed as follows: 2 min in running water, 4 min in Harris’ haematoxylin (Electron Microscopy Sciences), 10 s in 1% HCl in ethanol (acid‐alcohol), 3 min in dH_2_O, 30 s in tap water, 2 min in dH_2_O, 30 s in 1% eosin, then rehydrated via ethanol gradient and mounted with Pertex and cover slips. Periodic acid‐Schiff (PAS)/Alcian blue staining was performed as follows: 5 min in 1% Alcian blue solution + 3% acetic acid (Sigma) if using Alcian blue protocol, 1 min in dH_2_O, 5 min in 1% periodic acid (Thermo Fisher, 1 min in dH_2_O, 5 min in tap water, 1 min in dH_2_O, 15 min in Schiff's reagent (Sigma) if using PAS protocol, 1 min in dH_2_O, 5 min in tap water, 1 min in dH_2_O, 30 s in Meyer's haematoxylin (Sigma), 5 min in tap water, then rehydrated via ethanol gradient and mounted with Pertex and cover slips. Imaging was performed using an Axioimager M2 upright microscope (Zeiss). Images were processed and analysed using Fiji/ImageJ. Goblet cell counts per crypt and crypt length data were generated in Fiji for each mouse by counting the number of goblet cells (stained purple in H&E staining or blue in Alcian blue or PAS staining) per crypt, measuring the crypts’ length, using 6–20 crypts per mouse to generate each graphed data point.

### 4.6 RNAScope Staining

RNAscope staining (RNAscope Multiplex Fluorescent Reagent Kit v2, Biotechne) was performed on tissues fixed and sections as described above, according to the manufacturer's instructions. Briefly, following digestion with protease and hydrogen peroxide, and treatment in antigen retrieval buffer, sections were stained using the Co‐Detection Ancillary Kit with an anti‐EpCAM antibody (cat# ab71916, Abcam) for 2 h at RT. Sections were washed and then hybridised with *Tslp* or negative control probes (targeting a nonsense sequence) detection probes at 40°C. Probe sequences are proprietary. Sections were treated with amplification reagent, fluorophores conjugated (Opal 570, Akoya Biosciences), stained with a goat anti‐rabbit AlexaFluor 488 secondary antibody (cat#A‐11008, Invitrogen) for 90 min at RT, counterstained with DAPI, and mounted for image analysis. Imaging was performed using an Axioimager M2 upright microscope (Zeiss). Images were processed and analysed using Fiji/ImageJ.

### 4.7 RNA Extraction and cDNA Synthesis

Tissue harvested from mice directly, or processed as described in Enzymatic digestion of caecum, before being added to 1 mL of TRIzol (Thermo Fisher) in a 2 mL lysing matrix D beat bead mill‐compatible tube (MP Biomedicals). TRIzol stored tissues were thawed and homogenised using the Bead Mill 24 (Fisher Scientific) at 20 Hz for 4 min, then placed on ice. TRIzol stored cell suspensions were homogenised via pipetting before storage at −80°C. RNA extraction was performed using the Direct‐zol RNA Microprep Kit (Zymo Research). Briefly, TRIzol was added to a column and centrifuged at 14,000×*g* for 30 s, washed with the provided buffer, the RNA precipitated into the column matrix and repeatedly washed with buffer, before being eluted into 0.5 mL tubes (Eppendorf), washed with 75% ethanol, air dried after centrifugation, and solubilised in nuclease‐free water (Invitrogen). Sample RNA concentration was determined using the NanoDrop One (Thermo Fisher), and concentration adjusted downwards with nuclease‐free water if necessary. RNA was then stored at −80°C until reverse transcription could be performed. Reverse transcription reactions were performed using the Luna RT Supermix Kit (NEB), with the mass of RNA reverse transcribed kept consistent between samples within experiments. The resulting cDNA was stored at −20°C.

### 4.8 qPCR

qPCR reactions were performed on cDNA samples, produced as earlier described, using LunaScript Universal qPCR Master Mix (NEB) in 96‐well V‐bottomed 0.2 mL qPCR plates (ABI Fast Systems, Starlab). In short, 1 µL of sample was combined with 0.5 µL of forward primer, 0.5 µL of reverse primer (both at 10 mM), 4 µL of LunaScript master mix, and 14 µL of nuclease‐free water. Samples were run in triplicate, and the mean value taken for analysis, using three or two replicates (divergent single replicates were excluded if >1 CT difference from replicates was measured, or abnormal melt curves or amplification plots noted). Amplification was performed using the QuantStudio3 thermocycler (Thermo Fisher). Analysis was performed using the Thermo Fisher Connect Platform to visualise melt and amplification curves, and Microsoft Excel for the calculation of ΔCT and ΔΔCT values. ∆CT values were calculated using *Rpl13* transcript as a housekeeping gene, tested in the lab as producing more consistent CT values than alternatives *Bact* or *Gapdh* (data not shown). Melt curves and amplification plots were examined for all samples to validate primer binding and normal amplification. Calculation of 2^−ΔΔCT^ values used for graphing and analysis was performed by calculating ΔΔCT values by subtracting the control group's mean CT from each experimental group's CT, and calculating 2 to the power of this value's negative, to produce a “fold change” in transcript levels between control and experimental samples.

**Table:** 

Target transcript		Primer sequence (5’ > 3’)
*Rpl13*	F	CATGAGGTCGGGTGGAAGTA
	R	GCCTTTTCCGTAACCTCAA
*Tslp*	F	TTCACTCCCCGACAAAACAT
	R	GCCATTTCCTGAGTACCGTC
*Il33*	F	ATCACGGCAGAATCATCGAG
	R	GCGGTGCTGCTGAACTTT
*Il25*	F	AGCAGGGCCATCTCTCCT
	R	GTCTGTAGGCTGACGCAGTG

### 4.9 Enzymatic Digestion of Caecum

The caecum was dissected from mice, the contents scraped out, and the tissue washed with chilled HBSS ‐Ca/Mg (without Ca/Mg) (Sigma) by flushing followed by shaking in a 10 mL Falcon tube. Tissues were incubated in HBSS (‐Ca/Mg) +5 mM EDTA (Sigma) +1 mM DTT (Sigma) for 30 min at 37°C in a shaking incubator; the buffer was replaced halfway through the incubation. Tissues were then vortexed for 30 s, then passed through a 100 µm filter to isolate lamina propria from epithelial compartment single cell suspensions. At this point, the epithelial compartment and lamina propria were either transferred to separate lysing matrix D tubes for RNA extraction (see RNA extraction and cDNA synthesis) or the epithelial compartment was discarded, and the lamina propria was retrieved from the filter for further digestion. In the latter case, the lamina propria was minced with scissors and transferred into 5 mL of digest buffer consisting of HBSS with calcium and magnesium (Gibco) + 1% FCS +1 mg/mL of Liberase TL (Roche) +10 µg/mL DNAse (Sigma Aldrich). Tissue was incubated for 45 min at 37°C in a shaking incubator (mixed via pipetting at 15 and 30 min), then centrifuged at 400×*g* for 10 min at 4°C. Cells were resuspended in cold PBS, filtered using a 70 µm filter into a 10 mL tube, and centrifuged again. Cells were resuspended in chilled PBS, transferred into a V‐bottomed 96‐well plate (Corning), and kept at 4°C on ice. Cells were then stained using the PrimeFlow assay.

### 4.10 Enzymatic Digestion of Adipose Tissue for Flow Cytometry

The adipose tissue attached to the caecum was dissected and stored in chilled fat media (low glucose DMEM (Sigma) +5 g BSA (Melford) +25 mL HEPES (1 M) (Sigma). Samples transferred into digest buffer (per mouse, 3.5 mL fat media + 0.7 mg Liberase TL +0.875 mg DNase at 37°C for 30 min. 1.5 mL of fat media + 5 mM EDTA was added, tissue broken down by pipetting for 1 min, then transferred through a 70 µm filter into a 15 mL tube and centrifuged at 400×*g* for 10 min. Supernatant was removed, and cells were resuspended in cold PBS for staining. Cells were stained in chilled PBS using Zombie UV Fixable Viability Kit (BioLegend) at 1:1000 for 10 min at 4°C in the dark. Cells were washed (400×*g*, 5 min, 4°C) twice in chilled FACS buffer (PBS + 1% FCS), then stained using anti‐mouse antibodies for 20 min at 4°C at a 1:200 concentration in FACS buffer:

**Table:** 

Target	Conjugate	Clone (company)	Dilution	Stock conc. (µg/mL)
CD45	PE	I3/2.3 (BioLegend	1:200	1
CD140a (PDGFRα)	APC	APA5 (BioLegend)	1:200	1
CD16/32	None	93 (eBioscience)	1:200	1

### 4.11 Adipose Tissue Digest for MACS Sorting, Ex Vivo Culture, and ELISA

MACS sorting of adipose tissue was performed using the Adipose Tissue Progenitor Isolation Kit (Miltenyi Biotec) as per the manufacturer's instructions, using MidiMACS and MiniMACS separators. Sorted cells were plated at 10 × 10^5^ cells/mL for 24 h incubation at 37°C. Cells were transferred into 1.5 mL tubes, centrifuged at 14 000×*g* for 10 min at RT, and supernatant transferred into fresh 1.5 mL tubes for ELISA analysis. ELISAs were performed using the Biotechne Duoset TSLP ELISA (cat#DY555‐05), according to the manufacturer's instructions, using supernatant from stimulated cell lines or ex vivo cells. OD values were acquired using the VersaMax plate reader.

## Author Contributions

C.P.L. performed most of the experimental work, all analysis, and wrote the manuscript. S.T., E.P, and L.C. assisted in the experimental work, particularly mouse sample harvesting and technical advice. S.H.L. developed and provided the CCL24‐Cre mice for use in this project. R.K.G. acquired funding for, and conceptualised, the project, and assisted in the composition of the manuscript and figures.

## Conflicts of Interest

The authors declare no conflicts of interest.

## Supporting information




**Supporting File**: eji70248‐sup‐0001‐SuppMat.pdf.

## Data Availability

Raw data files are available from the corresponding author(s) upon reasonable request.
