# Supplementary material for: Caecal Epithelium‐Derived Thymic Stromal Lymphopoietin is not Required for Protective Immunity Against Whipworm
Source: Eur J Immunol. 2026 Jul 24;56(7):e70248. doi: 10.1002/eji.70248 (PMC13397033; doi:10.1002/eji.70248)
Supplement: Supplementary file 1 — Supporting File: eji70248‐sup‐0001‐SuppMat.pdf. [file EJI-56-e70248-s001.pdf]

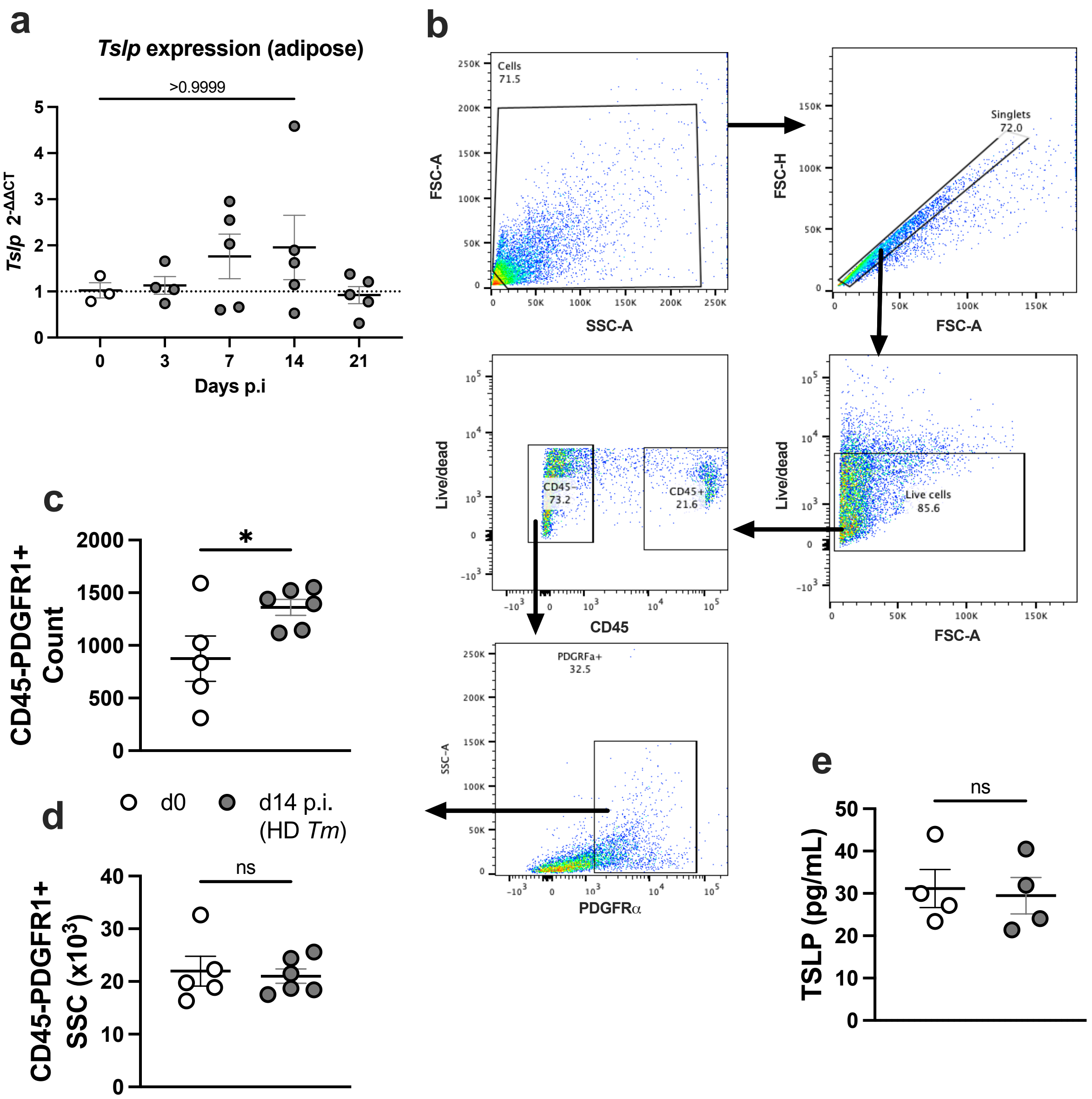
a
Tslp expression (adipose)
>0.9999
Tslp 2-ΔΔCT
Days p.i
b
Cells 71.5
FSC-A
SSC-A
Singlets 72.0
FSC-H
FSC-A
Live/dead
CD45- 73.2
CD45+ 21.6
CD45
Live cells 85.6
PDGFRa+ 32.5
SSC-A
PDGFRα
c
CD45-PDGFR1+ Count
*
d0
d14 p.i. (HD Tm)
d
CD45-PDGFR1+ SSC (x10³)
ns
e
TSLP (pg/mL)
ns

Supplementary Figure 1 (previous page) Alterations to caecum-associated adipose during Trichuris infection. (**a**) Mice were infected with 300 *Tm* eggs on day 0, and adipose tissue connected to the caecum was harvested and processed for RT-qPCR using given timepoints, with ΔCT values again generated against *Rpl13*, ΔΔCT values generated against day 0 ΔCT, and log transformed to give fold-change ($2^{-\Delta\Delta CT}$). (n=3-5 mice per group, Kruskal-Wallace testing). (b) Flow cytometry gating strategy for digested adipose tissue, with graphed CD45- PDGRFα+ cell count (**c**), and average side scatter of CD45-PDGFRα+ cells (**d**) (n=5-6, unpaired t test.) (**e**) TSLP detected *via* ELISA from the supernatant produced by mesenchymal adipose precursor cells isolated from caecal adipose *via* MACS column sorting and cultured for 24hrs (n=4, Mann-Whitney). All samples were generated from male C57BL/6 mice between 8 and 16 weeks old.

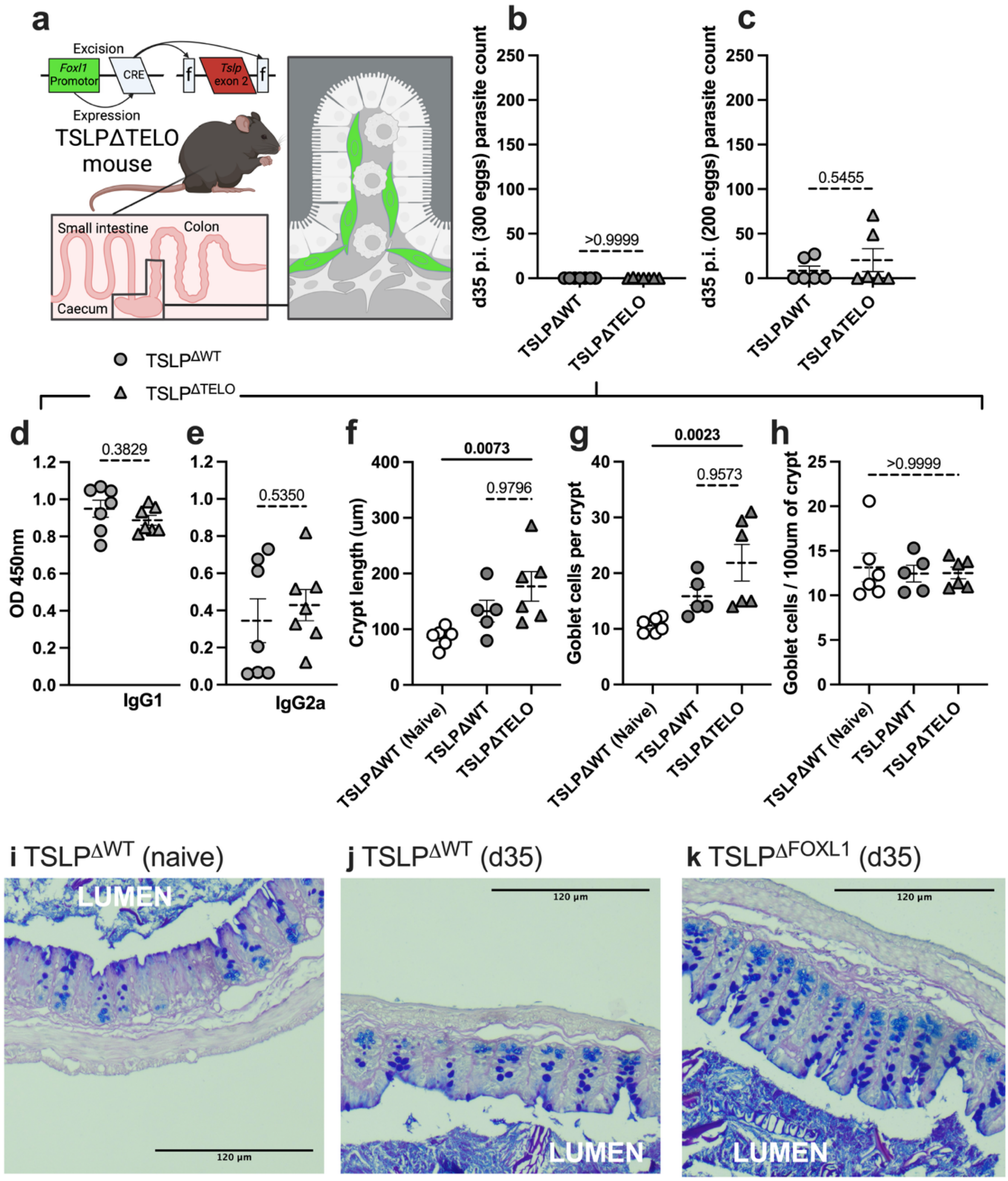
a
Excision
Foxl1 Promotor
CRE
f
Tslp exon 2
f
Expression
TSLPΔTELO mouse
Small intestine
Colon
Caecum
b
d35 p.i. (300 eggs) parasite count
>0.9999
TSLPΔWT
TSLPΔTELO
c
d35 p.i. (200 eggs) parasite count
0.5455
TSLPΔWT
TSLPΔTELO
TSLPΔWT
TSLPΔTELO
d
OD 450nm
0.3829
IgG1
e
0.5350
IgG2a
f
Crypt length (um)
0.0073
0.9796
TSLPΔWT (Naive)
TSLPΔWT
TSLPΔTELO
g
Goblet cells per crypt
0.0023
0.9573
h
Goblet cells / 100um of crypt
>0.9999
i TSLPΔWT (naive)
LUMEN
120 µm
j TSLPΔWT (d35)
120 µm
LUMEN
k TSLPΔFOXL1 (d35)
120 µm
LUMEN

Supplementary Figure 2 (previous page) Indications of immunity to HD *Tm* infection in TSLPΔTELO mice and littermate controls. (a) Generation and infection of conditional knockout mice schematic. **(b-c)** Counts of adult parasites recovered from TSLPΔTELO mice and WT littermates at d35 p.i. with 300 (**b**) or 200 (**c**) *Tm* eggs in two separate experiments. (n=6-7 mice per group, Mann-Whitney) (**d-h**) Further analysis performed on the mice infected in **b**. Serum level of parasite-specific IgG1a (**d**) and IgG2a/c (**e**), measured *via* ELISA and graphed in arbitrary optical density units (n=7 mice per group, Mann-Whitney). Length of caecal crypts (**f**), number of goblet cells per crypt (**g**), and goblet cells per 100ul of crypt (**h**) were measured *via* analysis of Alcian Blue/Periodic Acid-Schiff's histological staining of caecal sections (d35 p.i.) (**i-k**: representative images) in ImageJ (n=5-6 mice per group, Kruskal-Wallace). Each point used in f-h represents an average of at least six crypts from one mouse. Mice between 12 and 16 weeks old at time of harvest. Non-significant ($p>0.05$) comparisons are dashed.

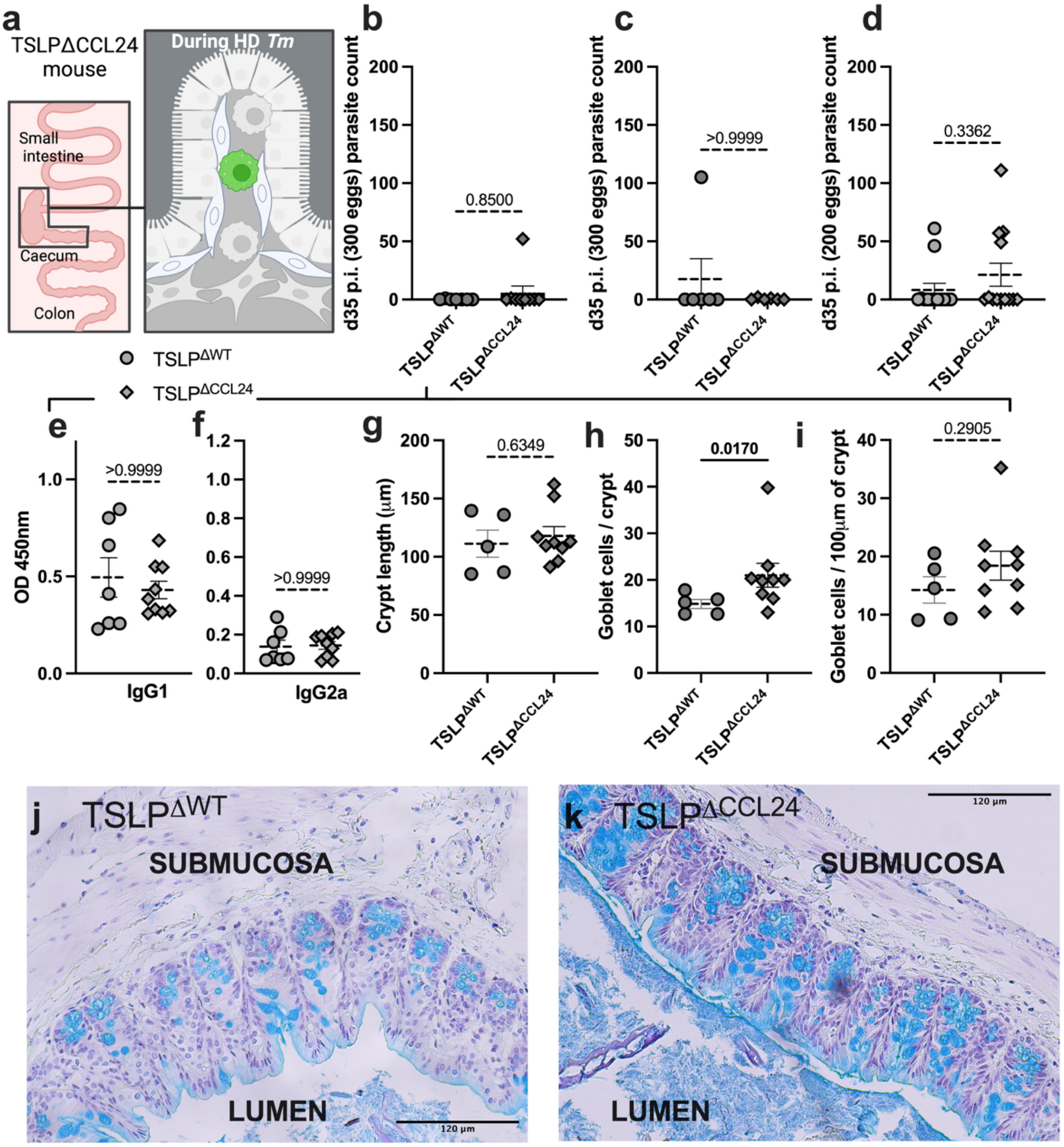

a
TSLPΔCCL24 mouse
During HD Tm
Small intestine
Caecum
Colon
TSLPΔWT
TSLPΔCCL24
b
d35 p.i. (300 eggs) parasite count
0.8500
c
d35 p.i. (300 eggs) parasite count
>0.9999
d
d35 p.i. (200 eggs) parasite count
0.3362
e
OD 450nm
>0.9999
IgG1
f
>0.9999
IgG2a
g
Crypt length (μm)
0.6349
h
Goblet cells / crypt
0.0170
i
Goblet cells / 100μm of crypt
0.2905
j TSLPΔWT
SUBMUCOSA
LUMEN
120 μm
k TSLPΔCCL24
SUBMUCOSA
LUMEN
120 μm

Supplementary Figure 3 (previous page) Indications of immunity to HD *Tm* infection in TSLPΔCCL24 mice and littermate controls. (a) Generation and infection of conditional knockout mice schematic. **b-c)** Counts of adult parasites recovered from caeca in ΔCCL24 mice and WT littermates infected with 300 *Tm* eggs, at d35 p.i. from two separate experiments (**d**) The experiment described in **b** and **c** was repeated substituting a 200-egg infection. **e-i**) Further analysis performed on the mice infected in **b**. Serum level of parasite-specific IgG1a (**e**) and IgG2a/c (**f**), measured *via* ELISA and graphed in arbitrary optical density units. Length of caecal crypts (**g**), number of goblet cells per crypt (**h**), and goblet cells per 100μm of crypt (**i**) were measured *via* analysis of Alcian blue (**j-k**: representative images) histological staining of caecal sections (d35 p.i.) in ImageJ (n=5-7 mice per group). Each point used in **f**-**h** represents an average of at least six crypts from one mouse. All data were tested *via* Mann-Whitney, excepting i (unpaired t test). Mice between 12 and 16 weeks old at time of harvest. Non-significant (p>0.05) comparisons are dashed.
